# Rapidly progressive glomerulonephritis and acute kidney injury associated with cocaine use - Case report

**DOI:** 10.1590/2175-8239-JBN-2020-0034

**Published:** 2020-06-22

**Authors:** Paulo Vitor de Souza Pimentel, Hermany Capistrano Freitas, Marcos Diógenes Braga Leite, Rafael Siqueira Athayde Lima, Dulce Maria Sousa Barreto, André Costa Teixeira, Elizabeth De Francesco Daher

**Affiliations:** 1 Universidade Federal do Ceará, Faculdade de Medicina, Fortaleza, CE, Brasil.; 2 Hospital Geral de Fortaleza, Departamento de Nefrologia, Fortaleza, CE, Brasil.; 3 Universidade Federal do Ceará, Faculdade de Medicina, Departamento de Medicina Clínica, Programa de Pós-Graduação em Ciências Médicas, Fortaleza, CE, Brasil.

**Keywords:** Glomerulonephritis, Cocaine, Acute kidney injury, Glomerulonefrite, Cocaína, Lesão renal aguda

## Abstract

A wide spectrum of renal complications can occur with acute and chronic use of cocaine. Most cases are related to rhabdomyolysis, but other mechanisms are malignant hypertension, renal ischemia, and rapidly progressive glomerulonephritis (RPGN) associated-ANCA vasculitis. In recent years, the use of cocaine adulterated with levamisole has been associated with ANCA vasculitis and pauci-immune RPGN. RPGN is clinically manifested as a nephritic syndrome with a rapid and progressive decline in renal function, and its histopathological finding is the presence of crescents in more than 50% of the glomeruli. We report a case of a 38-year-old man chronic user of cocaine, alcohol, and cigarettes who had red urine, oliguria, swollen legs and eyelids, as well as the uremic symptoms anorexia, emesis, and mental confusion. He was admitted with acute kidney injury and performed six hemodialysis sessions during the first 16 days of hospitalization and then was transferred to a tertiary hospital for diagnostic investigation. Tests of ANF (antinuclear factor), ANCA, anti-DNA, serology for hepatitis B, C, and HIV virus were negative. A renal percutaneous biopsy revealed crescentic glomerulonephritis with mild tubular atrophy. The patient underwent pulse therapy with methylprednisolone (for 3 days) and cyclophosphamide. Then he maintained daily prednisone and monthly intravenous cyclophosphamide and evolved with progressive improvement of renal function.

## RESUMO

Um amplo espectro de complicações renais pode ocorrer com o uso agudo e crônico de cocaína. A maioria dos casos está relacionada à rabdomiólise, mas outros mecanismos são hipertensão maligna, isquemia renal e glomerulonefrite rapidamente progressiva (GNRP) associada à vasculite por ANCA. Nos últimos anos, o uso de cocaína adulterada com levamisol tem sido associado à vasculite por ANCA e GNRP pauci-imune. A GNRP se manifesta clinicamente como uma síndrome nefrítica, com um declínio rápido e progressivo da função renal, e seu achado histopatológico é a presença de crescentes em mais de 50% dos glomérulos. Relatamos um caso de um homem de 38 anos, usuário crônico de cocaína, álcool e cigarros que apresentava urina vermelha, oligúria, pernas e pálpebras inchadas, além dos sintomas urêmicos, anorexia, êmese e confusão mental. Foi internado com lesão renal aguda e realizou seis sessões de hemodiálise durante os primeiros 16 dias de internação, e depois foi transferido para um hospital terciário para investigação diagnóstica. Os testes de ANF (fator antinuclear), ANCA, anti-DNA, sorologia para vírus da hepatite B, C e HIV foram negativos. Uma biópsia percutânea renal revelou glomerulonefrite crescente, com leve atrofia tubular. O paciente foi submetido à pulsoterapia com metilprednisolona (por 3 dias) e ciclofosfamida. Em seguida, ele manteve a prednisona diária e a ciclofosfamida intravenosa mensal e evoluiu com melhora progressiva da função renal.

## INTRODUCTION

A wide spectrum of renal complications can occur with the acute and chronic use of cocaine due to different renal hemodynamic and structural changes^1^. Acute kidney injury (AKI) is one of the most important complications from abusive use of cocaine and most cases are related to rhabdomyolysis^1^. Other mechanisms leading to AKI are the development of malign hypertension, renal ischemia, and rapidly progressive glomerulonephritis (RPGN) ANCA-associated vasculitis^1^.

RPGN is clinically manifested as a nephritic syndrome (hematuria, proteinuria, oliguria, edema, and hypertension) with rapid and progressive decline of renal function which may progress for end-stage renal failure if early treatment is not performed. The main histological finding is the presence of glomerular crescents in more than 50% of the glomeruli. Cocaine has been adulterated with levamisole, a nicotinic antagonist that potentiates the dopaminergic effects of cocaine and can induce pauci-immune RPGN and AKI^2^. The present study reports the case of a patient who underwent RPGN and AKI due to cocaine use.

## CASE REPORT

A 38-year-old man was admitted to an emergency department after intense alcohol consumption and cocaine use. He reported a nine-day history of oliguria, red urine, and swollen legs, abdomen and eyelids. He also presented abdominal pain, nausea, vomiting, and generalized myalgia, especially in the calf. 

He reported hypertension and irregular use of captopril. He had routine use of non-steroidal anti-inflammatory drugs (NSAIDs) for two years due to myalgia and toothache, smoking (23 packs/year), alcohol consumption (1L distillate drink/day) for 23 years and cocaine use for 20 years 3-4 times a week. He denied injection drug use.

On admission, his blood pressure was 150/95 mmHg. Heart and lung exams were normal. His abdomen was diffusely painful. The lower limbs were swollen. The laboratory findings were serum creatinine 12.97 mg/dL, serum urea 161 mg/dL, sodium 126 mEq/L, and potassium 5.5 mEq/L. The urinalysis showed proteinuria (+++) and hematuria. The proteinuria was 5,428 mg per 24 hours. Blood pressure was controlled with clonidine and amlodipine. Hemodialysis therapy was started due to symptoms of uremia and altered renal function. He was submitted to six hemodialysis sessions and then transferred to a tertiary hospital to pursue further etiological investigation.

**Figure 1:**
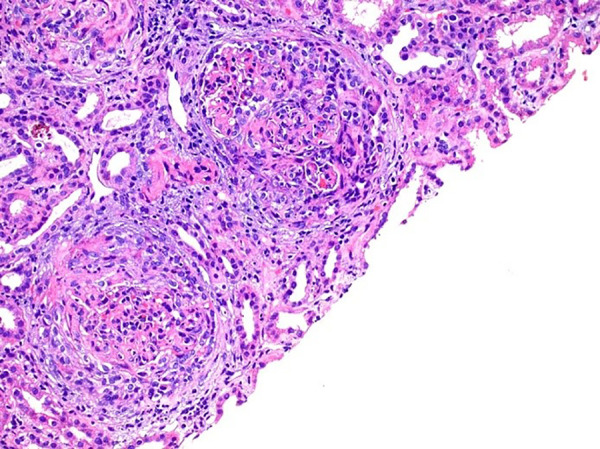
Cellular crescents, neutrophilic infiltrate, and segmental fibrinoid necrosis. Hematoxylin-eosin staining, 400x magnification.

**Figure 2:**
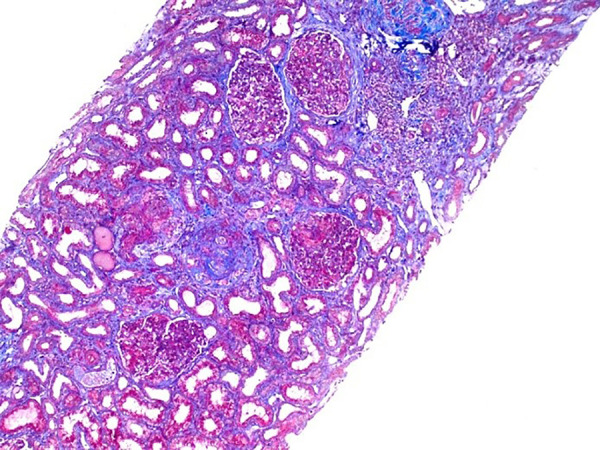
Mild interstitial fibrosis, global glomerulosclerosis. Masson's trichrome staining, 100x magnification.

After sixteen days in the first hospital, he was admitted to the nephrology referral hospital. On physical examination, blood pressure was 140/90 mmHg and heart rate was 86 bpm. Heart, lungs, abdomen and lower limbs were normal. The fundoscopic examination was normal. The laboratory data revealed serum creatinine 8.4 mg/dL, serum urea 145 mg/dL, sodium 137 mEq/L, and potassium 3.7 mEq/L. Further serum analysis showed creatine phosphokinase (CPK) 29 U/L, parathormone (PTH) 97.3 pg/mL, 25-hydroxy vitamin D 21.1 ng/mL, and phosphorus 5.5 mg/dL (Table 1). The serology tests for hepatitis B and C, HIV, and Chaga’s disease were negative. Venereal disease research laboratory (VDRL) test, antinuclear factor (ANF), anti-neutrophil cytoplasmic antibody (ANCA), and anti-double stranded DNA (anti-dsDNA) antibodies were negative. The transthoracic echocardiogram (TTE) and urinary tract ultrasound scan were normal. A renal percutaneous biopsy was performed and optical microscopy revealed a crescentic glomerulonephritis (10 glomeruli with cellular crescents formation and 1 glomerulus with fibrous crescent among 22 glomeruli studied) with mild tubular atrophy (Figures 1 and 2). Immunofluorescence microscopy showed granular capillary loop staining for C3c (++) and negative for C1q, IgM, IgA, IgG, kappa, and lambda. Pulse therapy with methylprednisolone 1 g/day (for 3 days) and cyclophosphamide was initiated. The patient presented improvement of renal function and hemodialysis was withdraw on the 13th day of hospitalization. He was discharged asymptomatic and the serum urea was 73 mg/dL, serum creatinine 1.5 mg/dL, serum albumin 3.8 g/dL, and urinalysis with proteinuria (+) and without red cells.

**Table 1:** Laboratory finding during hospitalization of a 38-year-old men with suspected rapidly progressive glomerulonephritis.

Day	1	3	6	12	18	20
Urea (mg/dL)	145	84	74	127	96	91
Creatinine (mg/dL)	8.4	5.4	4.3	3.4	1.9	1.7
Potassium (mEq/L)	3.7	3.8	3.4	5.0	4.9	4.9
Sodium (mEq/L)	137	137	136	135	137	136
Calcium (mg/dL)	-	9.0	9.1	8.6	8.4	-
Phosphorus (mg/dL)	-	5.5	4.4	4.5	5.4	-
Hematocrit (%)	23	-	24.6	21.1	24.5	26.4
Hemoglobin (g/dL)	8.2	-	8.7	7.3	8.6	8.6
White blood cells (x10^3^/mm^3^)	7.9	-	8.9	7.4	15.6	14.1
Platelets (x10^3^/mm^3^)	181	-	211	214	215	191
Direct bilirubin (mg/dL)	-	0.09	0.05	-	-	-
Indirect bilirubin (mg/dL)	-	0.3	0.24	-	-	-
Urine - Red blood cells	-	+++	+++	-	-	0
White blood cells	-	+	+	-	-	0
Protein	-	+++	++	-	-	+

## DISCUSSION

There are between 14 and 21 million cocaine users worldwide (0.3-0.5% of the population aged 15-64)^1^. In Brazil, prevalence rates suggest that the country is among the nations with the highest annual cocaine use^3^. AKI is one of the major cocaine abuse complications.

Acute and chronic use of cocaine is associated with nephrotoxicity related to changes in renal hemodynamics (vasoconstrictor effect) and structural changes mediated by increased oxidative stress, which induces changes in mesangial matrix synthesis and increased interstitial tubule fibrosis^4^^,^ 5.

Therefore, cocaine and its adulterants, especially levamisole, directly and indirectly contribute to renal function and structure alterations, mainly regarding microhemodynamic changes. Increased oxidative stress, platelet activation, production and activation of prostaglandins, sympathetic activity and endothelial dysfunction are the main pathophysiological effects caused by cocaine use. Consequently, the spectrum is varied and is divided into three groups: hypertension, chronic kidney disease, and acute kidney injury^6^. Cocaine use is related to other systemic damage, such as damage to the orbital cavity midline, necrosis of the hard and soft palate, pulmonary toxicity, hepatotoxicity, cardiovascular toxicity, and central nervous system toxicity^7^. Cocaine adulterated with levamisole is associated with retiform purpura, leukopenia and agranulocytosis, and alveolar hemorrhage^8^.

Most cocaine-associated AKI cases are related to the development of rhabdomyolysis and its pathophysiology is not fully understood. It involves one or more of the following factors: ischemia, hyperthermia, direct muscular toxicity of cocaine, and disseminated intravascular coagulation. Besides, a small portion of cocaine-associated AKI cases may occur without rhabdomyolysis evidence and are related to malignant hypertension development^9^^,^ 10. Although the pathophysiological process is also not fully understood, the proposed mechanisms include intra-renal vasoconstriction and ischemia, blockade of norepinephrine reuptake due to cocaine, and increased adrenal catecholamine release^9^^,^ 10.

The patient presented nephritic syndrome after alcoholic beverages and cocaine use, with a rapid progression to renal dysfunction. The renal dysfunction was characterized by severe AKI and uremia.

The patient was at higher risk for the development of renal complications due to the weekly cocaine consumption for 20 years. The hypothesis of AKI due to rhabdomyolysis was raised because of myalgia and cocaine abuse history. However, the absence of CPK measurement in the first hospital and the normal CPK value at the referral hospital made it difficult to establish this diagnosis. In addition, the absence of retinal lesions on fundoscopy, hypertensive encephalopathy, and renal biopsy findings reduced the probability of AKI associated with malignant hypertension. 

The renal percutaneous biopsy revealed a crescentic glomerulonephritis with mild tubular atrophy. RPGN is considered the most aggressive form of glomerulonephritis and represents a syndrome with clinical manifestation of a glomerulonephritis of rapid onset with progressive loss of renal function that may evolve to end-stage renal failure if treatment is not established^11^^-^ 13. 

Immunofluorescence was negative for immunoglobulin deposition, but ANCA was negative and the patient did not present clinical evidence of ANCA-associated vasculitis (microscopic polyangiitis, granulomatosis with polyangiitis, or eosinophilic granulomatosis with polyangiitis)^12^^,^ 14 ^,^ 15.

In recent years, cocaine has been adulterated with levamisole. This drug is used as a veterinary anthelmintic and as an immunomodulator in rheumatoid arthritis. Levamisole has been associated with ANCA vasculitis and pauci-immune RPGN. Serological analysis shows positivity for ANCA in 85% of the patients, with perinuclear pattern (p-ANCA) in 93%, and mixed p-ANCA and cytoplasmic (c-ANCA) patterns in 7%; 57% had antibodies against both myeloperoxidase (anti-MPO) and proteinase 3 (anti-PR3), and 43% had anti-MPO alone^8^.

In the present case, p-ANCA and c-ANCA analysis were performed twice with an interval of 45 days, both with negative results. Anti-MPO and anti-PR3 were not performed because they were unavailable in that hospital. Although not having laboratory data confirming the use of levamisole-adulterated cocaine, the presence of pauci-immune RPGN raised this hypothesis. 

The current approach for patients with RPGN is based on the combination of corticosteroids and cytotoxic drugs aimed at fighting inflammation, cellular response, and antibody production. Usual treatment is initiated with intravenous methylprednisolone followed by 1 mg/kg/day oral prednisone and intravenous (0.5-1g/m² dose) or oral (2-3mg/kg/24h) cyclophosphamide. Plasmapheresis may be used for the removal of circulating antibodies or immunocomplexes, as occurs in RPGN type I and in the presence of alveolar hemorrhage of RPGN type III^16^.

The patient received pulse therapy with 1g/day methylprednisolone for 3 days followed by daily treatment with 80 mg/day prednisone. In addition, a cyclophosphamide intravenous pulse therapy session was prescribed for 6 consecutives months. The initial dose of cyclophosphamide was 1250 mg, followed by an additional 500 mg monthly doses. However, the patient attended only two pulses, losing follow-up after 3 months. Despite this, in the last medical appointment, the treatment had improved the patient’s renal function and urinary output. Serum creatinine at the last visit was 0.8 mg/dL, urea was 31 mg/dL, and urinalysis with proteinuria was (++) and without red cells.

Due to the high prevalence of worldwide cocaine use, it is essential to know the potential complications of acute and chronic cocaine use. In addition to the otorhinolaryngological, cardiovascular, pulmonary, and neurological complications, it is important to remember the high risk of kidney injury. Among these complications, we should be aware of rhabdomyolysis, thrombotic microangiopathy, acute interstitial nephritis, renal infarction and pauci-immune RPGN occurrence. Therefore, given the widespread use of illicit drugs, physicians should include screening of cocaine use during RPGN investigation. 
